# Prevalence of Upper Extremity Musculoskeletal Disorders in Patients with Type 2 Diabetes in General Practice

**DOI:** 10.3390/medicines8020008

**Published:** 2021-02-01

**Authors:** Login Ahmed S. Alabdali, Jasmien Jaeken, Geert-Jan Dinant, Marjan van den Akker, Bjorn Winkens, Ramon P. G. Ottenheijm

**Affiliations:** 1 Department of Family Medicine, CAPHRI Care and Public Health Research Institute, Maastricht University, P.O. Box 616, 6200 MD Maastricht, The Netherlands; geertjan.dinant@maastrichtuniversity.nl (G.-J.D.); m.vandenAkker@allgemeinmedizin.uni-frankfurt.de (M.v.d.A.); ramon.ottenheijm@maastrichtuniversity.nl (R.P.G.O.); 2 Ministry of Education, Riyadh 12435, Saudi Arabia; 3 Department of Public Health and Primary Care, Catholic University of Leuven, Kapucijnenvoer 33, B-3000 Leuven, Belgium; j.jaeken@alumni.maastrichtuniversity.nl; 4 Institute of General Practice, Goethe University, Frankfurt am Main, 60323 Frankfurt, Germany; 5 Academic Centre for General Practice/Department of Public Health and Primary Care, KU Leuven, Naamsestraat 80, B-3000 Leuven, Belgium; 6 Department of Methodology and Statistics, CAPHRI Care and Public Health Research Institute, Maastricht University, P.O. Box 616, 6200 MD Maastricht, The Netherlands; bjorn.winkens@maastrichtuniversity.nl

**Keywords:** type 2 diabetes, musculoskeletal disorders, prevalence, diabetes complication

## Abstract

**Background:** One of the lesser recognized complications of diabetes mellitus are musculoskeletal (MSK) complications of the upper and lower extremity. No prevalence studies have been conducted in general practice. Thus, the aim of this study was to investigate the prevalence of upper extremity MSK disorders in patients with type 2 diabetes (T2DM) in the Netherlands. **Methods:** We conducted a cross-sectional study with two different approaches, namely a representative Dutch primary care medical database study and a questionnaire study among patients with T2DM. **Results:** In the database study, 2669 patients with T2DM and 2669 non-diabetes patients were included. MSK disorders were observed in 16.3% of patients with T2DM compared to 11.2% of non-diabetes patients (*p* < 0.001, OR 1.53, 95% CI 1.31, 1.80). In the questionnaire study, 200 patients with T2DM were included who reported a lifetime prevalence of painful upper extremity body sites for at least four weeks of 67.3%. **Conclusion:** We found that upper extremity MSK disorders have a high prevalence in Dutch patients with T2DM presenting in general practice. The prevalence ranges from 16% based on GP registered disorders and complaints to 67% based on self-reported diagnosis and pain. Early detection and treatment of these disorders may play a role in preventing the development of chronic MSK disorders.

## 1. Introduction

Diabetes (DM) has many well-known and well-understood complications, such as diabetic retinopathy, nephropathy, and polyneuropathy, which are currently screened for during the periodic check-ups [1]. However, studies have shown that DM is also associated with an increased prevalence of numerous musculoskeletal (MSK) disorders [2,3,4,5], including shoulder, hand, and lower extremity disorders [6,7,8], which are currently not screened for. 

The pathophysiological mechanism of MSK disorders is not fully understood, but evidence suggests that increased accumulation of advanced glycation end-products (AGEs) plays an important role [1,9]. AGEs are formed by the non-enzymatic condensation of metabolic intermediates and glucose, and this process is increased or stimulated in chronic hyperglycemia. AGE accumulation occurs in connective tissue causing damage to the tendons, joint capsule, ligaments and nerves, which leads to structural and functional deterioration. AGE formation leads to collagen disposition in the periarticular connective tissue causing the damage [9,10,11]. 

Several international studies have shown that MSK disorders have an increased prevalence in T2DM. For example, the prevalence of frozen shoulder ranges from 5% to 30% in patients with DM and from 2% to 5% in patients without DM [6]. The wide range in observed prevalence in patients with DM might be caused by differences in used study methods, i.e., database and questionnaire studies. Moreover, patients with T2DM have higher odds if developing MSK disorders compared to patients without DM (OR 1.1, 95% CI:1.0, 1.3), and age seems to be associated: patients with DM aged <60 years have a higher odd ratio than those without DM aged <60 years (OR 1.6, 95% CI:1.2, 2.2) [12]. 

Inadequate management of MSK disorders leads to a decrease in functional ability, an increasingly inactive lifestyle and a poorer quality of life; factors that undermine DM treatment [13,14]. Therefore, it is important to diagnose and treat MSK disorders at an early stage. Even though international studies have already concluded that physical examination of the hand and shoulder should be included in the evaluation of DM patients [15], this is currently not implemented in the Dutch nor international guidelines. To better inform developers of guidelines and policymakers, the next step is to investigate the prevalence of MSK disorders in the Netherlands. In the well-organized Dutch healthcare system, almost all patients with T2DM are treated by care groups composed of general practitioners (GPs) and trained diabetes nurses. Routine check-up visits including screening for complications take place at least once a year [16]. Therefore, it is questionable if the prevalence is as high as reported in the international literature.

### Objectives

Our cross-sectional study aims to investigate the prevalence of overall and specific upper extremity MSK disorders in patients with T2DM in the Netherlands using two approaches, namely (1) a primary care medical database study and (2) a questionnaire that was handed out only to patients with T2DM attending their routine DM check-ups in general practice. The reason for combining two methods instead of selecting just one is that a much better insight into the MSK prevalence and its potential modifiers can be achieved. 

## 2. Methods 

We carried out a cross-sectional study composed of the two different approaches. The study was approved by the Medical Ethics Committee of Zuyderland Medical Centre (METC-Z 17-T-138, date of approval: 23 November 2017).

### 2.1. Approach 1 (RNFM Database)

We used data from the Research Network Family Medicine (RNFM), which is a large anonymized medical database of a GP network in the Maastricht University region, the Netherlands. RNFM was developed in 1988 and reflects the national healthcare system where patients are registered with a GP and access all healthcare through their GP. The network consists of 65 GPs from 22 GP practices. RNFM is composed of computerized medical data of approximately 105,000 patients (reference year 2017). Current and relevant past health problems (i.e., diseases, diagnoses and prescribed drugs of all patients) are recorded systematically and updated continuously along with the basic sociodemographic characteristics of the patients. Registration of these medical data is part of daily routine in the participating GP practices, and every three months registered health problems are added and uploaded to the RNFM database [17,18]. The International Classification of Primary Care (ICPC) is used to code and register health problems. Next to the ICPC code for T2DM (T90.02), the following ICPC codes were used to define upper extremity MSK disorders: Shoulder symptoms/complaints (L08), Shoulder syndromes (L92), Wrist symptoms (L11), Hand/finger symptoms/complaints (L12), Carpal tunnel syndrome (CTS) (N93), Dupuytren’s contracture (L99.03), Trigger finger (L99.04), Rheumatoid arthritis and related conditions (L88), and Osteoarthritis (L91). However, there is no ICPC code for MSK. All patients with T2DM aged between 18 and 70 years old who were registered in January 2017 were included and matched with patients without any type of DM to compare the difference in prevalence. Matching was in a ratio of 1:1, based on age, gender, and general practice [19]. Additionally, a maximum age of 70 was chosen because we believe that MSK disorders in patients older than 70 years are more likely based on age related degenerative processes. 

### 2.2. Approach 2 (Questionnaire in General Practice)

This approach was carried out in the Meditta region, the Netherlands. Meditta is a company organized by GPs in the area of Sittard-Geleen, Roermond and Weert. Part of their activities includes providing T2DM care in the so-called diabetes care group. In this region, T2DM care is delivered by specially trained diabetes nurses employed by Meditta, who work in GP practices under supervision of the GP. T2DM care in the Netherlands is usually delivered by GPs and specially trained diabetes nurses. During a six month-period, T2DM patients between 30 and 70 years of age were approached upon visiting their diabetes nurse during their annual routine check-ups and were asked to fill in a questionnaire inquiring about MSK pain and medical history.

Additionally, to increase the recruitment, an announcement was placed on the website of the Dutch Diabetic Association (*Diabetesvereniging Nederland*, DVN) and in their periodic *Diabetes Magazine*. 

All included patients withT2DM received the questionnaire during their check-up, and the diabetes nurse filled in a short case report form per patient, containing a last reading of HbA1C, the current body mass index (BMI) and year of T2DM diagnosis. Patients were asked to fill in the questionnaire and send it to our research center in the provided return envelope.

The outcome measures were categorized into a complaint and disease level. On a complaint level, the following subcategories were distinguished: point prevalence of painful body sites (shoulder, elbow, wrist, hand or fingers), lifetime prevalence of painful body sites, prevalence of the most painful body site, and on a diagnosis level: prevalence of specific MSK disorders (frozen shoulder, CTS, trigger finger and Dupuytren’s contracture). We defined point prevalence as the proportion of patients with T2DM with pain at time of filling in the questionnaire while lifetime prevalence was defined as the proportion of patients with T2DM who has suffered from pain any time in the past for at least 4 weeks.

The questionnaire was compiled on the basis of existing ones:

- *The Douleur Neuropathique 4 questionnaire (DN4) [20,21,22,23]*: A commonly used questionnaire for screening and diagnosing neuropathic pain in patients with neurological complaints, valid for the Dutch population and validated to be used in patients with DM. For our study purpose, we left out the physical examination questions, and only used the two interview questions, which composed of 7 items (yes/no answers). The cut-off point for neuropathic pain is considered to be 4 out of 7 points.
- *Pain questionnaire [24]*: A six-item questionnaire that classifies chronic MSK pain is adapted from the version used in epidemiological research by the Arthritis Research Campaign in the United Kingdom, translated in Dutch, and previously used in the Maastricht Study.
- *Epidemiology of diabetes intervention and complications association questionnaire for cheiroarthropathy [25]*: This questionnaire was used to assess the medical history of upper extremity MSK disorders (yes/no answers). We incorporated eight questions concerning history of symptoms and previous diagnosis while the examination part was excluded. These questions were translated from English to the Dutch language.
- Self-reported comorbidity. A list of diseases derived from two sources was used; lists of the Study of Medical Information and Lifestyles in Eindhoven (SMILE), and the National Institute for Public Health and the Environment (RIVM) [26,27].

### 2.3. Sample Size Calculation

In this calculation, we have assumed a prevalence of 12.8% for frozen shoulder [28], and prevalence for specific MSK disorders starting from 5–15% for trigger finger [6] up to 20% for frozen shoulder [29]. Therefore, for approach 2, we used a conservative expected proportion of 0.12. Assuming a 10% response rate, we have handed over 1900 questionnaires to the diabetes nurses. This results in an expected number of 190 patients. By using a conservative expected proportion of 0.12, the width of the corresponding 95% CI is then equal to about 0.09, i.e., a 95% CI of 0.07 to 0.16. For approach 1, all patients with T2DM will be included, which is expected to exceed this number of 190 patients, implying an accurate estimate of the prevalence of MSK disorders.

### 2.4. Data Analysis 

#### 2.4.1. Approach 1: RNFM Database

Proportions were calculated for the prevalence of overall and specific upper extremity MSK disorders in DM patients and non-diabetes. Patients with T2DM were compared with non-diabetes using logistic regression correcting for the matching variables age, gender and general practice. Additionally, for patients with T2DM, logistic regression was used to assess which of the variables age, gender, duration of DM, rheumatoid arthritis and osteoarthritis were independently related to the outcomes of MSK in general and specific MSK disorders. 

#### 2.4.2. Approach 2: Questionnaire Study

For patients with T2DM in this approach, proportions were calculated for both overall and specific upper extremity point and lifetime prevalence of painful body sites on a complaint level, and for specific MSK disorders on diagnosis level. Logistic regression analyses were used to assess which variable (age, gender, duration of T2DM, body mass index (BMI), HbA1C, rheumatoid arthritis, osteoarthritis and other joint inflammation) was independently related to the outcomes of MSK in general and specific MSK disorders. Interaction between several variables, suggested in the literature or who have possible biological influences, were tested. 

For both approaches, linearity assumption for numerical variables was assessed by testing whether a quadratic centered term improved the model fit significantly. In case the linearity assumption was violated, the analysis was repeated using a categorized variable (including dummy variables) instead of linear and quadratic terms, where these results were compared with those with linear and quadratic terms to see whether the same quadratic trend was represented by both analyses. The numerical variables were categorized using cut-off values based on the number of subjects per category and on sensible values, for example 50, 55, 60, and 65 years for age in approach 1 or 25, 30, and 40 for BMI [30]. Multicollinearity was checked using variance inflaction factors (VIF), where VIF >10 indicate a collinearity problem, and influential outliers were defined as Cook’s distance > 1. 

Odds ratios (OR) with corresponding 95% confidence intervals (CI) and two-sided *p*-values were reported, where *p*-values ≤ 0.05 were considered statistically significant. Statistical analyses were performed using IBM SPSS Statistics for Windows (version 25.0, Armonk, NY, USA).

## 3. Results

### 3.1. Approach 1: RNFM Database

#### 3.1.1. Study Population Characteristics 

A total of 2669 patients with T2DM and 2669 patients without DM were included. Mean age was 60.3 years, ranging from 20–70 years, and a proportion of 40.1% females. The duration of T2DM was on average 8.6 years and ranged from 0 to 47 years (Table 1). 

#### 3.1.2. MSK Disorders Overall

MSK disorders were observed in 16.3% (CI:14.8,17.7) of T2DM compared to 11.2% (CI:10.0,12.5) of non-diabetes patients (Table 2).

#### 3.1.3. Shoulder Complaints and Syndromes

The prevalence of any shoulder disease in patients with and without DM was 9.2% (CI:8.1, 10.3) and 6.6% (CI:5.7, 7.6), respectively (Table 2).

#### 3.1.4. Hand Complaints or Syndromes

The prevalence of any hand disease in patients with and without DM was 9.7% (CI:8.6, 10.9) and 6% (CI:5.1, 6.9), respectively. The highest prevalence of a specific MSK disorder was trigger finger which was present in 3.2% (CI:2.6, 4.0) of patients with T2DM and in 1.2% (CI:0.8, 1.7) of non-diabetes patients. The prevalence of CTS and Dupuytren’s contracture are presented in Table 2. 

#### 3.1.5. Regression Analyses and Associations

Duration of DM and age were both categorized in five subgroups, as linearity assumption was violated for these variables, where the results reported in Table 3 were similar as those with linear and quadratic terms. Odds for overall MSK disorders increased significantly with duration of DM (OR ranges from 3.02 to 5.7) being significantly higher for age-categories above 55 years compared to 50 years or younger (OR ranges from 1.5 to 2.1), and this was significantly higher for females than males (OR 1.3, CI:1.1, 1.7). See Table 3 for more details.

### 3.2. Approach 2: Questionnaire Study

#### 3.2.1. Study Population Characteristics

In total, two hundred patients with T2DM have completed the questionnaire: 182 patients were recruited via the diabetes nurses and 18 via the Diabetes Magazine. The mean age was 61.7 years, proportion of females was 39.2%, mean duration of DM was 9.5 years, mean BMI was 29.9 kg/m^2^, and mean HbA1C was 55.3 mmol/mol. At the time of filling in the questionnaire, 100 patients (50%) reported to have pain in the upper extremity of whom 46.5% were females (Table 1).

#### 3.2.2. Prevalence on Complaint Level

##### I—Life-Time Prevalence 

A total of 67.3% (*n* = 134) of patients with T2DM reported that they have had painful body site for at least 4 weeks, and 46.6% (*n* = 89) reported to have had any shoulder complaints. Additionally, 34.5% (*n* = 69) specifically reported that they had been suffering from a painful stiff shoulder in their lifetime.

##### II—Point Prevalence 

The shoulder was the most prevalent painful site (*n* = 78, 39%), and 22% (*n* = 44) reported to have unilateral shoulder pain. Hand or fingers, elbow, upper arm, wrist, and forearm pain was reported in 30% (*n* = 61), 13.6% (*n* = 27), 7% (*n* = 14), 5% (*n* = 10) and 4% (*n* = 8), respectively.

Regarding the prevalence of the most painful affected body sites, we observed that among patients with pain for at least 4 weeks (*n* = 200), the shoulder was the most prevalent painful body site (*n* = 43/189, 22.8%), and 16.5% (*n* = 33) reported to have unilateral pain. 

Neuropathic pain was reported in 34.8% (*n* = 15/43) of those patients. Hand and fingers pain, and wrist pain was reported in 26.7% (*n* = 24/90), and 3.3% (*n* = 3/90), respectively, and of those patients, 29% (*n* = 7/24) and (*n* = 2/3) reported neuropathic pain (Table 4).

#### 3.2.3. Prevalence on Diagnosis Level

CTS was the most prevalent disorder (55.5%, *n* = 106/200), followed by frozen shoulder and Dupuytren’s contracture, both with 41% (*n* = 79). Trigger finger was reported in 39.8% (*n* = 76) (Table 4). 

#### 3.2.4. Regression Analyses and Associations

Interactions did not add significantly to the models (*p*-values ranged from 0.127 to 0.672). The multivariable analyses showed that rheumatoid arthritis and osteoarthritis were significantly associated with shoulder pain point prevalence (OR 4.5, CI: 1.3, 14.6 and OR of 3.1, CI:1.5, 6.2, respectively), frozen shoulder (OR 3.3, CI:1.1, 9.9 and OR 2.3, CI:1.1, 4.7, respectively). In addition, rheumatoid arthritis was significantly associated with trigger finger (OR 7.0, CI:1.8, 27.8), whereas osteoarthritis was significantly associated with CTS (OR 4.2, CI:2.0, 8.8) and Dupuytren’s contracture (OR 3.1, CI:1.5, 6.2). Furthermore, for every year of increase in age, the odds of developing Dupuytren’s contracture increases significantly (OR 1.06, CI:1.006, 1.1), while the odds of developing a frozen shoulder decreases significantly (OR 0.9, CI:0.8, 0.9). Additionally, duration of T2DM is associated with shoulder pain (OR 1.08, CI:1.01, 1.1 per year increase) and females have higher odds of developing CTS (OR 2.2, CI:1.1, 4.6) (Table 5). All results of the univariate analyses are presented in Supplementary Table S1.

## 4. Discussion 

This is the first study evaluating the prevalence of upper extremity MSK disorders in patients with T2DM in general practice. This study was conducted with two different approaches and showed a prevalence for MSK disorders in patients with T2DM ranging from 16% based on by GP registered disorders and complaints (database study) to 67% based on self-reported diagnosis and pain (questionnaire study). This difference can be explained by the nature of the two approaches. The medical database study contains data of patients with T2DM who sought medical attention for their MSK disorders, otherwise these disorders would not have been registered by the GP, while in the questionnaire study, patients were attending their regular DM check-up and not primary seeking medical attention for MSK complaints. Therefore, it is plausible that the results of the database study might be an underestimation of the real prevalence and the questionnaire study might have caused an overestimation, as patients with T2DM suffering from pain might be more eager to participate. In this approach, half of the patients reported to have pain at time of filling in the questionnaire. 

Additionally, we observed that the shoulder is the most affected body site in both studies, and that age, duration of T2DM, and gender show conflicting, statistically significant associations between the studies, except for the duration of T2DM and shoulder disorders/complaints, females, and CTS, which show a statistically significant positive association in both studies. 

### 4.1. Comparison with the Literature

When comparing the observed prevalence with international studies, we noticed that there are no studies conducted in general practice. A cross-sectional study conducted in an outpatient diabetes centre in the USA reported a prevalence of shoulder pain with or without disability in 63% of the patients with DM. This result was obtained by using the Shoulder Pain and Disability Index (SPADI). This is a higher prevalence compared to the observed 39% in our questionnaire study. This difference may be explained by the fact that we only asked for shoulder pain and not for disability, and that our population consistent only of patients with T2DM [31]. A tertiary hospital-based study conducted in Pakistan investigating MSK disorders of the upper limb extremity using a survey and physical examination, showed prevalences somewhere between our database and questionnaire studies [32]. The differences can be explained by the difference in study settings and healthcare system. Another cross-sectional population-based study conducted in Norway, that also used a questionnaire approach, reported a prevalence of chronic MSK complaints of 58% in patients with T2DM, which is approximately 9% lower than observed in our study [12]. They defined MSK complaints as pain and/or stiffness ≥3 months during the last year, where we used a duration of ≥4 weeks, which might explain the difference between the two studies. A hospital-based study conducted in Turkey where patients were physically examined showed a prevalence of frozen shoulder in patients with T2DM of 13% and 1.3% for CTS [28], which is much lower than we observed. This large difference might be explained by the study design; we calculated lifetime prevalence in our questionnaire study, while in the hospital-based study, the point prevalence is estimated in patients having shoulder pain at the time of consultation. Therefore, we can conclude that study design, setting, healthcare system, and definition of MSK disorders might influence prevalence.

### 4.2. Strengths and Limitations

The RNFM database contains medical data representative for the Dutch population [18], and made it possible to compare the prevalence of MSK disorders in patients with T2DM and patients without DM, which are major strengths of this work. GPs affiliated with the RNFM register the data of their patients, including ICPC codes, in a uniform manner and meet twice per year for training. 

To overcome the problem of using only a single approach to determine the prevalence, we also performed a questionnaire study, which has two main advantages. First, unregistered MSK disorders and complaints that are missed in database studies can be included, because patients did not report them to the GP or because the GP judged them to be clinically not significant. Second, it enabled us to include BMI, HbA1C, rheumatoid arthritis, osteoarthritis, and other joint inflammation disorders in the analysis. However, a disadvantage is that recruiting patients for a questionnaire study might result in selection bias or reporter bias.

Despite the large sample size in the database study, the number of patients with T2DM diagnosed with rheumatoid arthritis and osteoarthritis was too small to correct for in the regression analysis, and additionally, we were unable to test the influence of BMI and HbA1C as this information was not registered in the database yet, which can be seen as limitations. Additionally, there might be other confounding factors associated with diabetes and MSK disorders, including medication, depression and health services use. Although statins may cause MSK pain in patients with T2DM, yet all patients with T2DM in The Netherlands actually are advised to always use statins, which is why we did not report about number of patients using this medication. Regarding co-morbidities, we have chosen to select only the most relevant ones. Unfortunately, we were unable to correct for the volume of health services used as this is not registered in the RNFM database. 

Last, we have chosen a 1:1 matching between patients with T2DM and patients without DM, while other proportions were possible. However, we expected to have enough power of the study using 1:1 matching, which is proven by the statistical significance of the results found. 

### 4.3. Clininal Implications and Future Research

In the context of clinical practice, our findings indicate that MSK disorders have a high prevalence in patients with T2DM and that screening for these disorders seems to be advisable. We define screening as a protocolled history taking and focused physical examination addressing MSK disorders during periodic regular DM check-up visits. Early detection and treatment of these disorders may play a role in preventing the development of chronic disorders, which might negatively influence DM treatment. However, to better inform guideline and policymakers, it would be useful to conduct a trial to investigate the effectiveness in two study groups, one with MSK screening and early management incorporated and a second group without. 

## 5. Conclusions

We found that registered and non-registered upper extremity MSK disorders have high a prevalence in Dutch patients with T2DM presenting in general practice, and that prevalence is influenced by study design and definition of MSK disorders. The prevalence ranges from 16% based on by GP registered disorders and complaints (database study) to 67% based on self-reported diagnosis and pain (questionnaire study). The early detection and treatment of these disorders may play a role in preventing the development of chronic disorders. Screening for these disorders seems advisable, although policymakers might require a trial investigating the effectiveness.

## Figures and Tables

**Table 1:** Study population demographic data of the two different approaches in Dutch general practice: (RNFM approach *n* = 5338, Questionnaire approach *n* = 200).

	RNFM Study		Questionnaire Study		
Characteristics	Patients with Type 2 Diabetes	Patients without Diabetes	Total	Patients with Type 2 Diabetes with Pain Complaints When Filling the Questionnaire	Patients with Type 2 Diabetes without Pain Complaints When Filling the Questionnaire
	*n* = 2669	*n* = 2669	*n* = 200	*n* = 100	*n* = 100
Age					
mean ± SD	60.3 ± 8.1	60.3 ± 8.1	61.7 ± 6.8	61.5 ± 6.6	61.9 ± 6.9
range (years)	20–70	20–70	32–70	38–70	32–70
Females %	40.10%	40.10%	39.2%*	46.5% *	32.00%
Duration of diabetes					
mean ± SD	8.6 ± 6.2	NA	9.5 ± 6.3	10.1 ± 6.9	8.9 ± 5.5
range (years)	0–47		0–39	0–39	0–26
BMI (kg/m^2^)					
mean ± SD	NA	NA	29.9 ± 5.7	30.2 ± 6.5	29.7 ± 4.8
range			16.0–58.8	16.0–58.8	19.4–44.9
HbA1C (mmol/mol)					
mean ± SD	NA	NA	55.3 ± 11.3	55.7 ± 12.3	54.9 ± 10.3
range			39.0–131.0	39.0–131.0	40.0–93.0
Rheumatoid arthritis					
*n*	10/2669	8/2669	22/195 *	19/97 *	3/98 *
(%)	(0.40)	(0.30)	(11.30)	(19.50)	(3.10)
Osteoarthritis					
*n*	24/2669	59/2669	82/196 *	56/98 *	26/98 *
(%)	(0.90)	(2.20)	(41.80)	(57.10)	(25.80)

RNFM, Research Network Family Medicine; Numerical data are presented as mean ± standard deviation (SD) and range, Nominal data as number of patients (percentage). NA: not applicable. * Missing data.

**Table 2:** Prevalence of specific upper extremity musculoskeletal disorders and their corresponding ICPC codes in patients with type 2 diabetes compared to patients without diabetes by logistic regression in RNFM approach, correcting for the matching variables age, gender and general practice.

ICPC Code	Patients with Type 2 Diabetes	Patients without Diabetes	OR (95% CI); *p*-Value
	*n*	*n*	
	(%)	(%)	
	95% CI	95% CI	
MSK overall (any ICPC code of the chosen codes)	434/2669	300/2669	1.55 (1.3, 1.8); <0.001
	(16.30)	(11.20)	
	14.8, 17.7	10.0, 12.5	
Any shoulder disease (ICPC: L08 or L92)	242/2639	175/2639	1.41 (1.1, 1.7); 0.001
	(9.20)	(6.60)	
	8.1, 10.3	5.7, 7.6	
Shoulder complaints (ICPC: L08)	154/2573	102/2573	1.53 (1.1, 1.9); 0.001
	(6.00)	(4.00)	
	5.1, 6.9	3.2, 4.8	
Shoulder syndromes (ICPC: L92)	115/2491	84/2491	1.38 (1.04, 1.8); 0.023
	(4.60)	(3.40)	
	3.8, 5.5	2.7, 4.1	
Any hand disease (ICPC L11, L12, N93, L99.03 or L99.04)	260/2669	160/2669	1.71 (1.3, 2.1); <0.001
	(9.70)	(6.00)	
	8.6, 10.9	5.1, 6.9	
Wrist complaints (ICPC: L11)	40/2639	21/2639	1.83 (1.08, 3.1); 0.023
	(1.50)	(0.80)	
	1.1, 2.0	0.5, 1.2	
Hand/finger complaints (ICPC: L12)	98/2620	71/2620	1.42 (1.0, 1.9); 0.024
	(3.70)	(2.70)	
	3.0, 4.5	2.1, 3.4	
CTS (ICPC: N93)	52/2525	27/2525	2.02 (1.2, 3.2); 0.003
	(2.10)	(1.10)	
	1.5, 2.7	0.7, 1.5	
Dupuytren’s contracture (ICPC: L99.03)	38/2654	22/2654	1.79 (1.1, 3.0); 0.030
	(1.40)	(0.80)	
	1.0, 1.9	0.5, 1.2	
Trigger finger (ICPC: L99.04)	85/2626	32/2626	2.81 (1.8, 4.2); <0.001
	(3.20)	(1.20)	
	2.6, 4.0	0.8, 1.7	

RNFM, Research Network Family Medicine; CI, confidence interval; OR, odds ratio; CTS, carpal tunnel syndrome; MSK disorders, musculoskeletal disorders.

**Table 3:** Regression analysis of specific upper extremity musculoskeletal disorders in patients with type 2 diabetes in RNFM approach ^a^.

	Overall MSK Disorders	Any Shoulder Disorder	Shoulder Complaints	Shoulder Syndrome	Any Hand Disease	Wrist Complaints	Hand/Finger Complaints	Trigger Finger	Dupuytren’s Contractor	CTS
	(Any ICPC Code of the Chosen Codes)	(ICPC: L08 or L92)	(ICPC: L08)	(ICPC: L92)	(ICPC L11, L12, N93, L99.03 or L99.04)	(ICPC: L11)	(ICPC: L12)	(ICPC: L99.04)	(ICPC: L99.03)	(ICPC: N93)
	OR (95% CI); *p*-Value	OR (95% CI); *p*-Value	OR (95% CI); *p*-Value	OR (95% CI); *p*-Value	OR (95% CI); *p*-Value	OR (95% CI); *p*-Value	OR (95% CI); *p*-Value	OR (95% CI); *p*-Value	OR (95% CI); *p*-Value	OR (95% CI); *p*-Value
Age in years										
≤50	Reference	Reference	Reference	Reference	Reference	Reference	Reference	Reference	Reference	Reference
51–55	1.5 (0.9–2.5); 0.098	1.5 (0.8–2.8); 0.197	1.3 (0.6–2.8); 0.444	2.5 (1.1–6.2); 0.039	1.4 (0.7–2.8); 0.258	0.8 (0.2–3.5); 0.836	0.5 (0.1–2.3); 0.421	2.8 (0.7–10.7); 0.116	1.3 (0.2–8.3); 0.723	2.7 (0.8–8.7); 0.087
56–60	2.1 (1.3- 3.4); 0.001	1.8 (1.01–3.2); 0.033	1.9 (1.0–3.8); 0.048	1.6 (0.7–4.0); 0.246	2.1 (1.2–3.8); 0.008	1.9 (0.6–6.0); 0.242	0.6 (0.2–2.2); 0.507	3.3 (0.9–11.5); 0.057	1.3 (0.2–6.8); 0.740	4.3 (1.5–12.5); 0.007
61–65	2.1 (1.3–3.3); 0.001	1.7 (1.0–3.1); 0.039	1.6 (0.8–3.2); 0.128	1.7 (0.7–4.1); 0.179	2.2 (1.2–3.9); 0.005	1.2 (0.3–3.8); 0.748	1.1 (0.4–3.4); 0.738	5.0 (1.5–16.8); 0.008	2.3 (0.5–10.9); 0.259	3.1 (1.08–9.1); 0.035
66–71	1.7 (1.1–2.6); 0.015	1.3 (0.7–2.2); 0.322	1.0 (0.5–2.0); 0.908	1.7 (0.7–3.8); 0.200	1.8 (1.03–3.1); 0.037	1.4 (0.4–4.2); 0.524	0.8 (0.2–2.3); 0.680	2.4 (0.7–8.1); 0.153	2.5 (0.5–11.1); 0.213	2.4 (0.8–7.1); 0.093
Females	1.3 (1.1–1.7); 0.002	1.2 (0.9–1.5); 0.164	1.1 (0.8–1.5); 0.523	1.2 (0.8–1.8); 0.187	1.6 (1.2–2.1); 0.000	3.4 (1.9–6.2); 0.000	2.3 (1.2–4.4); 0.009	1.7 (1.1–2.6); 0.016	0.8 (0.4–1.6); 0.596	1.7 (1.1–2.5); 0.008
Duration of diabetes in years										
0–2	Reference	Reference	Reference	Reference	Reference	Reference	Reference	Reference	Reference ^b^	Reference
3–5	3.02 (1.7–5.2); 0.000	2.9 (1.3–6.4); 0.006	4.5 (1.5–12.9); 0.005	1.8 (0.5–5.8); 0.290	3.5 (1.7–7.3); 0.001	4.1 (0.5–34.0); 0.184	1.4 (0.2–7.6); 0.640	2.9 (0.8–10.3); 0.088	Reference ^b^	3.4 (1.1–10.1); 0.023
6–8	3.5 (2.08–6.1); 0.000	3.7 (1.7–8.0); 0.001	5.1 (1.8–14.8); 0.002	2.3 (0.7–7.1); 0.131	3.8 (1.8–7.9); 0.000	5.0 (0.6–40.4); 0.130	2.9 (0.6–13.7); 0.171	3.7 (1.08–12.7); 0.037	2.4 (0.8–6.9); 0.090	2.2 (0.7–6.7); 0.162
9–11	4.8 (2.8–8.3); 0.000	6.0 (2.8–12.9); 0.000	7.1 (2.5–20.3); 0.000	6.4 (2.2–18.4); 0.000	4.8 (2.3–9.9); 0.000	11.5 (1.5–88.0); 0.018	5.1 (1.1–23.2); 0.031	3.1 (0.9–11.1); 0.072	2.5 (0.8–7.2): 0.079	3.6 (1.2–10.8); 0.018
≥12	5.7 (3.3–9.6); 0.000	6.6 (3.1–13.8); 0.000	7.7 (2.7–21.5); 0.000	7.2 (2.5–20.2); 0.000	5.7 (2.8–11.6); 0.000	10.7 (1.4–80.9); 0.021	2.7 (0.5–12.6); 0.201	6.1 (1.8–20.1); 0.003	3.1 (1.2–8.1); 0.020	4.5 (1.6–13.0); 0.004

^a^ Rheumatoid arthritis and osteoarthritis were not included in these analyses due to low number of patients with those conditions. ^b^ The first two groups were combined as from 0–2 years in duration of diabetes were no patients with Dupuytren’s contractor. RNFM, Research Network Family Medicine; OR, odds ratio; CI, confidence interval; CTS, carpal tunnel syndrome.

**Table 4:** Prevalence of specific upper extremity musculoskeletal disorders on complaint or diagnosis level in patients with type 2 diabetes in the questionnaire approach.

Pain Prevalence	(*n*)	%	95% CI
***Pain Life time prevalence per body site, n = 200***			
Any painful body site for at least 4 weeks (Shoulder, arm, elbow, hand or fingers)	134 ^a^	67.3	61.0, 75.0
Any shoulder complaints	89 ^b^	46.6	39.6, 54.0
Painful stiff Shoulder	69	34.5	28.4, 42.1
***Pain Point prevalence per body sites, n = 200 ^a^***			
Shoulder: any side *	78	39.2	32.3, 46.0
unilateral	44	22	16.2, 27.9
Elbow: any side *	27	13.6	8.7, 18.3
unilateral	21	10.6	6.2, 14.8
Wrist: any side *	10	5	1.9, 8.0
unilateral	6	3	0.6, 5.4
Upper arm: any side *	14	7	3.4, 10.6
unilateral	8	4	1.2, 6.7
Forearm: any side *	8	4	1.2, 6.7
unilateral	6	3	0.6, 5.4
Hand (including fingers): any side *	61	30.7	24.1, 37.1
unilateral	26	13	8.3, 17.7
***Prevalence of*** ***the most painful affected body sites, n = 200 ^c^***			
Shoulder any side *	43	22.8	16, 28
unilateral	33	16.5	26, 46
Hand (including fingers)	24	12.7	17, 35
Wrist	3	1.6	−0.02, 3.3
***Prevalence of specific MSK disorders, n = 200***			
Frozen shoulder	79 ^b^	41.4	33.5, 48.2
Carpal tunnel syndrome	106 ^b^	55.5	48.8, 63.6
Trigger finger	76 ^b^	39.8	30.8, 45.3
Dupuytren’s contracture	79 ^d^	41.1	34.6, 49.4

Data presented in total number bilaterally (unilateral side), percentages and 95% confidence interval. ^a^ One is missing; ^b^ 9 are missing; ^c^ 11 are missing; ^d^ 8 are missing. * Any side is referred to either right or left side or both.

**Table 5:** Multivariable logistic regression analysis of specific upper extremity musculoskeletal disorders on complaint or diagnosis level in patients with type 2 diabetes in the questionnaire approach.

	Shoulder Pain Point Prevalence OR (95% CI); *p*-Value *n* = 78/200	Frozen Shoulder OR (95% CI); *p*-Value *n* = 79/200	Carpal Tunnel Syndrome OR (95% CI); *p*-Value *n* = 106/200	Trigger Finger OR (95% CI); *p*-Value *n* = 76/200	Dupuytren’s Contracture OR (95% CI); *p*-Value *n* = 79/200
	*p*-Value	*p*-Value	*p*-Value	*p*-Value	*p*-Value
	*n* = 78/200	*n* = 79/200	*n* = 106/200	*n* = 76/200	*n* = 79/200
Age in years	0.9 (0.9, 1.0); 0.144	0.9 (0.8, 0.9); 0.005	0.9 (0.9, 1.0); 0.441	1.03 (0.9, 1.1); 0.204	1.06 (1.006, 1.0); 0.031
Female	1.5 (0.7, 3.0); 0.250	1.04 (0.5, 2.0); 0.902	2.2 (1.1, 4.6); 0.023	1.2 (0.6, 2.6); 0.468	1.09 (0.5, 2.2); 0.802
Duration of diabetes	1.07 (1.0, 1.1); 0.015	1.03 (0.9, 1.1); 0.291	1.04 (0.9, 1.1); 0.216	1.03 (0.9, 1.1); 0.200	1.005 (0.9, 1.1); 0.848
BMI	0.9 (0.9, 1.1); 0.943	Cat 1: Ref * Cat 2: 0.3(0.1–0.9);0.036 *Cat 3*: 0.4(0.1–1.2);0.121 *Cat 4*: 0.2(0.4–1.3);0.099	0.9 (0.9, 1.1); 0.845	1.05 (0.9, 1.1); 0.104	1.04 (0.9, 1.1); 0.106
HbA1C	0.9 (0.9, 1.0); 0.793	0.9 (0.9, 1.0); 0.293	1.01 (0.9, 1.0); 0.504	0.9 (0.9, 1.0); 0.588	1.02 (0.9, 1.1); 0.102
Rheumatoid arthritis	4.5 (1.4, 14.6); 0.012	3.7 (1.2, 11.4); 0.022	0.5 (0.1, 2.0); 0.383	7.0 (1.8, 27.8); 0.005	1.5 (0.5, 4.5); 0.417
Other joint inflammation	1.2 (0.5, 2.9); 0.625	1.07 (0.4, 2.5); 0.861	2.8 (1.1, 7.4); 0.033	1.7 (0.7, 4.3); 0.189	1.02 (0.4, 2.4); 0.961
Osteoarthritis	3.1 (1.5, 6.2); 0.001	2.5 (1.2, 5.1); 0.011	4.2 (2.0, 8.8); <0.001	1.5 (0.7, 3.0); 0.236	3.1 (1.5, 6.2); 0.001

* Dummy variable was included to fix the linearity assumption violation. Cat 1: BMI 18.5 to 24.9; Cat 2:25 to 29.9; Cat 3:30 to 39.9; Cat 4: ≥40.

## Data Availability

The data presented in this study are available on request from the corresponding author.

## Supplementary Materials

The following are available online at https://www.mdpi.com/2305-6320/8/2/8/s1, Table S1: Crude model of Regression analysis of specific upper extremity musculoskeletal disorders on complaint or diagnosis level in type 2 diabetes patients in the questionnaire approach.
