# Supplementary material for: Prevalence of Upper Extremity Musculoskeletal Disorders in Patients with Type 2 Diabetes in General Practice
Source: Medicines (Basel). 2021 Feb 1;8(2):8. doi: 10.3390/medicines8020008 (PMC7912777; doi:10.3390/medicines8020008)
Supplement: Supplementary file 1 [file medicines-08-00008-s001.pdf]

medicines

Supplementary Materials: Prevalence of Upper Extremity Musculoskeletal Disorders in Patients with Type 2 Diabetes in General Practice

Login Ahmed S. Alabdali, Jasmien Jaeken, Geert-Jan Dinant, Marjan van den Akker, Bjorn Winkens, Ramon P. G. Ottenheijm

Table 1. Crude model of Regression analysis of specific upper extremity musculoskeletal disorders on complaint or diagnosis level in type 2 diabetes patients in the questionnaire approach.

	**Shoulder Pain Point Prevalence OR (95%CI); P-value** *n* **= 78/200**	**Frozen Shoulder OR (95%CI); P-value** *n* **= 79/200**	**Carpal Tunnel Syndrome OR (95%CI); P-value** *n* **= 106/200**	**Trigger Finger OR (95%CI); P-value** *n* **= 76/200**	**Dupuytren's Contracture OR (95%CI); P-value** *n* **= 79/200**
Age in years	1.001 (0.9, 1.04) 0.975	0.9 (0.9, 1.002) 0.060	0.9 (0.9, 1.03) 0.761	1.05 (1.005, 1.1) 0.029	1.06 (1.01, 1.1) 0.011
Females	1.6 (0.9, 2.9) 0.092	1.2 (0.7, 2.2) 0.428	2.2 (1.2, 4.2) 0.008	1.4 (0.7, 2.6) 0.226	1.2 (0.6, 2.1) 0.542
Duration of diabetes	1.07 (1.02, 1.1) 0.005	1.02 (0.9, 1.07) 0.269	1.04 (0.9, 1.09) 0.068	1.03 (0.9, 1.08) 0.176	1.02 (0.9, 1.07) 0.239
BMI	1.02 (0.9, 1.07) 0.281	*Cat 1: Ref** *Cat 2*: 0.3 (0.1-0.9); 0.045 *Cat 3*: 0.6(0.2-1.6); 0.353 *Cat 4*: 0.5(0.1-2.3); 0.395	1.02 (0.9, 1.07) 0.413	1.04 (0.9, 1.09) 0.130	1.04 (0.9, 1.09) 0.109
HbA1C	1.003 (0.9, 1.02) 0.799	0.9 (0.9, 1.01) 0.493	1.01 (0.9, 1.03) 0.439	0.9 (0.9, 1.01) 0.448	1.01 (0.9, 1.04) 0.193
Rheumatoid arthritis	5.1 (1.8, 13.7) 0.001	3.5 (1.3, 9.2) 0.009	1.2 (0.4, 3.1) 0.677	6.0 (2.0, 17.2) 0.001	2.8 (1.1, 7.0) 0.028
Other joint inflammation	2.5 (1.2, 5.2) 0.008	1.9 (0.9, 4.0) 0.055	2.8 (1.2, 6.2) 0.010	2.7 (1.3, 5.7) 0.005	1.7 (0.8, 3.6) 0.103
Osteoarthritis	4.0 (2.2, 7.4) <0.001	2.5 (1.4, 4.7) 0.002	4.2 (2.2, 8.0) <0.001	2.2 (1.2, 4.0) 0.009	4.0 (2.2, 7.5) <0.001

*Dummy variable was included to fix the linearity assumption violation. Cat 1: BMI 18.5 to 24.9; Cat 2:25 to 29.9; Cat 3:30 to 39.9; Cat 4: ≥ 40.
